# Molecular Epidemiology and Genetic Diversity of *Enterocytozoon bieneusi* in Cervids from Milu Park in Beijing, China

**DOI:** 10.3390/ani12121539

**Published:** 2022-06-14

**Authors:** Qingxun Zhang, Zhenyu Zhong, Zhiqiang Xia, Qinghui Meng, Yunfang Shan, Qingyun Guo, Zhibin Cheng, Peiyang Zhang, Hongxuan He, Jiade Bai

**Affiliations:** 1 Beijing Milu Ecological Research Center, Beijing 100076, China; zqx@milupark.org.cn (Q.Z.); zhyzh@milupark.org.cn (Z.Z.); xia414540@163.com (Z.X.); mengqinghui2006@163.com (Q.M.); shanyunfang@yeah.net (Y.S.); guoqingyun1987@126.com (Q.G.); czb@milupark.org.cn (Z.C.); 2 Henan Institute of Science and Technology, Xinxiang 453003, China; 3 National Research Center for Wildlife Borne Diseases, Institute of Zoology, Chinese Academy of Sciences, Beijing 100101, China; zhangpeiyang@ioz.ac.cn

**Keywords:** *Enterocytozoon bieneusi*, prevalence, genetic diversity, zoonosis, deer, China

## Abstract

**Simple Summary:**

*Enterocytozoon bieneusi* is the most prevalent microsporidian species that can cause significant intestinal diseases in both humans and animals worldwide. This study investigated the overall *E. bieneusi* prevalence of 21.9% (47/215) in captive and free-ranging deer species in Beijing, China. Thirteen *E. bieneusi* genotypes including six known and seven novel genotypes were identified. These resources will provide the insights to understand the veterinary and public health and the transmission dynamics between animal environments and human ones.

**Abstract:**

*Enterocytozoon bieneusi* is the most prevalent microsporidian species that can cause zoonotic diseases in humans and animals. Despite receiving increasing attention in relation to domestic animals, there has been limited information on the infection burden of *E. bieneusi* in cervids. Altogether, 215 fecal samples collected from four deer species in Beijing, China were examined by nested- Polymerase Chain Reaction (PCR)targeting the internal transcribed spacer (ITS) region. The overall prevalence of *E. bieneusi* in deer was 21.9% (47/215), with 30.0% (24/80) in Pere David’s deer, 27.3% (15/55) in fallow deer, 12.5% (5/40) in sika deer, and 7.5% (3/40) in Chinese water deer. Thirteen *E. bieneusi* genotypes were identified, including six known (HLJD-V, MWC_d1, BEB6, CGC2, JLD-XV, and HND-I) and seven novel genotypes (BJED-I to BJED-V, BJFD, and BJCWD). A phylogenetic analysis showed that 38.3% of the isolates belonged to zoonotic Group 1. In addition, *E. bieneusi* infection was first detected in fallow deer and Chinese water deer, which could act as potential zoonotic reservoirs. Our findings suggest that *E. bieneusi* circulates in deer and might be of importance to public health.

## 1. Introduction

*Enterocytozoon bieneusi* is an intestinal zoonotic fungus-like parasitic protist that can cause significant intestinal diseases in both humans and animals worldwide [1,2]. *E. bieneusi* infection, which is responsible for the majority of cases of human microsporidiosis, can cause persistent diarrhea, especially in immunocompromised individuals [3,4]. According to epidemiological investigations on *E. bieneusi* infections among human, the positivity rate of *E. bieneusi* was 2–78% in HIV-seropositive diarrheal patients, while it was 1.4–4.6% in HIV-seronegative patients [2]. In addition, microsporidiosis is common in children, and the recovery of *E. bieneusi* spores from children’ stools were associated with different clinical spectra (asymptomatic, diarrhea, or HIV-seropositive) [2]. *E. bieneusi* was transmitted by fecal-oral route, primarily through the consumption of food and water contaminated by infective spores or oocysts [5]. Currently, effective treatment of *E. bieneusi* infection is very limited, and only a few drugs have shown anti-microsporidian activity [6].

Due to the difficulty related to microscopic investigations, genotypes of *E. bieneusi* have been identified by analyzing the ribosomal internal transcribed spacer (ITS) sequences [7,8]. Globally, *E. bieneusi* consists of eleven groups (Group 1–11) and more than 500 genotypes based on ITS sequence analysis. Among these groups, Group 1 was considered to be the zoonotic genotypes, with genotypes A, D, EbpC, type IV, Peru6, Peru8, and Peru11 from Group 1a being the most prevalent genotypes identified in humans, which have also been frequently documented in domestic and wild animals [9]. Group 2 were once considered to be bovine-specific; however, genotypes I, J, BEB4, and BEB6 from Group 2 have also been found in other animals and in humans [5,10]. Group 3–11 were host-specific groups and were mainly isolated from dogs, rodents, deer, carnivores, wild animals, and water sources. Only several genotypes from Group 5, 6, 10 have been found in humans, suggesting a limited zoonotic potential. Compared to the high prevalence of Group 1 genotypes in pigs, ruminants were infected predominantly by Group 2 genotypes, and the infection rates vary widely around the world [11].

Recent epidemiological findings revealed the host expansion and interspecies infection of *E. bieneusi* in different wildlife, including captive or free-ranging populations [12,13,14,15]. Although the molecular characterization and genotypes analysis of *E. bieneusi* have been identified in deer, information regarding *E. bieneusi* in different deer species (especially Fallow deer and Chinese water deer) and regions is also limited. In the present study, we further investigated the molecular epidemiology, genotypes, and zoonotic potential of *E. bieneusi* infection in captive and free-ranging deer species in Beijing, China. Our results could provide the basic data for expanding the host range of *E. bieneusi* and preventing and controlling microsporidiosis in domestic animals and humans.

## 2. Materials and Methods

### 2.1. Specimen Collection

A total of 215 fecal samples were collected from different deer species at Beijing Père David’s deer Park between November and December 2021, including 80 samples from free-ranging Pere David’s deer (*Elaphurus davidianus*), 55 from captive Fallow deer (*Dama dama*), 40 from captive Sika deer (*Cervus nippon*), and 40 from free-ranging Chinese water deer (*Hydropotes inermis inermis*). For free-ranging animals, fecal samples were collected at the feeding point after artificially increasing the feeding, and the gender of the animals was determined by sex determining factor (SRY) gene using the PCR method [16]. All the fresh droppings were collected and transported to the laboratory following cold chain rules.

### 2.2. DNA Extraction and Nested PCR Amplification

The TIANamp Stool DNA Kit (TIANGEN BIOTECH, Beijing, China) was used to extract the genomic DNA from 200 mg fecal samples according to the manufacturer’s protocol. The extracted DNA was stored at −20 °C until PCR analysis. For the screening of *E. bieneusi* genotypes, nested PCR assays were used to amplify the internal transcribed spacer (ITS) gene, as described in our previous study [17]. Briefly, nested PCR was performed using the primers EBITS3 (5′-GGTCATAGGGATGAAGAG-3′) and EBITS4 (5′-TTCGAGTTCTTTCGCGCTC-3′) for the first amplification. The cycling conditions were as follows: 94 °C for 3 min for denaturation, and 35 cycles of denaturation at 94 °C for 30 s, followed by 55 °C for 30 s, 72 °C for 30 s, and a final extension at 72 °C for 10 min. The second reaction was performed using the primers EBITS1 (5′-GCTCTGAATATCTATGGCT-3′) EBITS2.4 (5′-ATCGCCGACGGATCCAAGTG-3′), which amplified a fragment of approximately 390 bp under the same PCR conditions as above. Positive *E. bieneusi* genotype BEB6 isolated from cattle and negative controls were included in all PCR tests. All secondary PCR products were electrophoresed on 1% agarose gel stained with GoldView.

### 2.3. DNA Sequence Analysis

All positive PCR products were bi-directionally sequenced by TSINGKE (Beijing, China). To identify *E. bieneusi* genotypes, the obtained sequences were firstly subjected to BLAST searches and aligned with reference sequences using MAFFT [18]. A phylogenetic analysis was performed using the neighbor-joining method as implemented in MEGA X [19], which was calculated by the Kimura 2-parameter model with 1000 bootstrap replicates. The obtained nucleotide sequences in this study have been deposited in GenBank under accession number OM876242-OM876258.

### 2.4. Statistical Analysis

The data were grouped into different variables in terms of species, gender, and living condition. Differences in the infection rates of each group were statistically calculated using the Chi-square test in SPSS 25.0. A *p*-value of <0.05 was considered significant.

## 3. Results

### 3.1. Occurrence of E. bieneusi

Of the 215 fecal samples examined, 47 (21.9%) samples were positive for *E. bieneusi* infection by PCR amplification. The prevalence of *E. bieneusi* varied between animal sources from 7.5% to 30.0%. The highest prevalence was in Pere David’s deer (24/80, 30.0%), followed by fallow deer (15/55, 27.3%), sika deer (5/40, 12.5%), and Chinese water deer (3/40, 7.5%) (Table 1). Mixed infections between genotypes were not found. Notably, a significantly higher prevalence of *E. bieneusi* was observed in Pere David’s deer and fallow deer than in sika deer and Chinese water deer (*p* < 0.05). However, no correlation between the gender and living condition was found (Table 2).

### 3.2. Distribution and Genetic Characterization of E. bieneusi

All positive PCR specimens were successfully sequenced to determine the genotypes of *E. bieneusi*. The genotyping analysis revealed the presence of 13 different genotypes, including six known genotypes (HLJD-V, MWC_d1, BEB6, CGC2, JLD-XV, and HND-I) and seven novel genotypes (named BJED-I to BJED-V, BJFD, and BJCWD), which were firstly identified in three deer species. The most prevalent *E. bieneusi* genotype was HLJD-V (*n* = 15), followed by BJFD (*n* = 10), MWC_d1 (*n* = 5), BEB6 (*n* = 3), and CGC2 (*n* = 3) (Table 1). The results also show that the genotypes were distributed in the four animal species differently. Genotype HLJD-V was found in Pere David’s deer, fallow deer, and Chinese water deer, while BJFD and CGC2 existed mainly in fallow deer and sika deer, respectively. Compared with genotypes in GenBank, the sequences of the novel genotypes both included 2–3 nucleotide mutations or deletions (Table 3).

### 3.3. Phylogenetic Analysis

**Figure 1:**
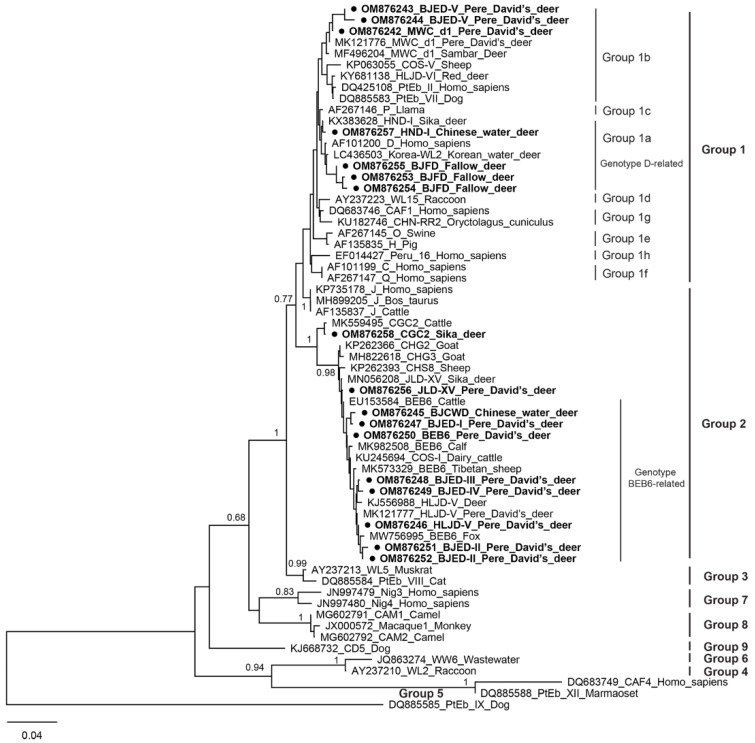
Phylogenetic relationships of ITS nucleotide sequences of *E. bieneusi* identified from deer in the present study and reference genotypes in GenBank. The genotypes identified in this study were indicated by a filled circle (●) and highlighted in bold.

Seventeen sequences (including one sequence each from type HLJD-V, MWC_d1, BEB6, CGC2, JLD-XV, HND-I, BJCWD, and BJED-I/III/IV; two sequences from BJED-II, two sequences from BJED-V, three sequences from BJFD) were included for the phylogenetic inference. The phylogenetic analysis showed that seven isolates recovered from Pere David’s deer, fallow deer, and Chinese water deer fell into the human pathogenic Group 1 and that the other 10 isolates were clustered into the bovine-specific Group 2 (Figure 1). Genotype BJFD was clustered together with Korea-WL2 (isolated from Korean water deer) in Group 1a (Genotype D-related). Genotype BJMD-V showed a higher sequence identity with MWC_d1 and was clustered within Group 1b. Other novel genotypes were mainly clustered into Group 2 and showed a higher similarity with BEB6, and they were considered to be Genotype BEB6-related genotypes.

## 4. Discussion

Microsporidiosis, caused by *E. bieneusi*, is an important emerging infectious parasite in humans and animals. Wildlife including a captive or free-living population has been regarded as an environmental reservoir and the zoonotic origins of *E. bieneusi* [12]. Currently, the general view is that frequent contact of humans with domestic animals and wildlife has been recognized as an important risk factor for the zoonotic transmission of *E. bieneusi* [9,20]. To explore the role of wild animals in the epidemiology of microsporidiosis, we characterized for the first time the prevalence and genotype of *E. bieneusi* in captive and free-ranging deer species in Beijing, China.

We observed an overall prevalence of 21.9%, with infection rates ranging from 7.5% to 30.0% in samples from four deer species. In view of the molecular epidemiological studies of *E. bieneusi* infection in deer worldwide, the infection rate varied drastically from 4.1% to 53.6% (Table 4). A relatively lower infection rate of 4.1% and 16.8% was reported in wild sambar deer and reindeer in Australia [15] and the northeast of China [21], while a higher infection rate of 32.5% and 53.6% was reported in wild white-tailed deer [22] and Korean water deer [12]. For Pere David’s deer, the prevalence of *E. bieneusi* in the present study (30.0%) correlated with other reports published in the Henan (34.0%) and Hubei (35.2%) province of China [23,24]. For farmed deer, the prevalence was 7.1–35.9% in sika deer, red deer, Siberian roe deer, and Forest musk deer (Table 4), and our study observed a prevalence of 12.5% in captive sika deer. In two studies conducted in China and Australia, *E. bieneusi* was found to be negative in a small number of fallow deer [15,25]. To the best of our knowledge, *E. bieneusi* infection was first identified in fallow deer (27.3%), which suggested that fallow deer could act as potential reservoirs. Water deer include two subspecies: Chinese water deer (*Hydropotes inermis inermis*) and Korean water deer (*Hydropotes inermis argyropus*). Compared with the 53.6% *E. bieneusi* infection rate in wild Korean water deer [12], our study firstly reported a lower infection rate of 7.5% in free-ranging Chinese water deer. Our research identified that the prevalence of *E. bieneusi* infection was significantly associated with deer species, probably because of the different susceptibilities of different species. In addition, the differences in infection rate in deer may be potentially attributed to many factors such as the various living conditions, biogeographic distributions, age, and health status of animals [5,17].

High genotypic heterogeneity and phenotypic diversity has been revealed for *E. bieneusi* using ITS genotyping analysis with 11 phylogenetic groups identified in humans, livestock, wild animals, birds, and water sources worldwide. Genotypes in Group 1, 2 have been found in a broad range of hosts and are probably responsible for most zoonotic transmissions of *E. bieneusi* infections [9]. Ruminants including both domestic and wild animals are common reservoir hosts of these genotypes [1,8]. To date, more than 60 *E. bieneusi* ITS genotypes have been identified in different deer species worldwide. Deer are major hosts of genotypes in Group 2 such as I-, J-, BEB6-, and HLJD-associated genotypes, and they can also carry some genotypes in Group 1 such as D, MWC_d1, Type IV, EbpC, and Peru6 (Table 4). Our study identified 13 distinct genotypes, including six known and seven novel genotypes. The new genotypes were determined based on previous studies, which produced sequences with three nucleotide substitutions [12,32]. Among these genotypes, Group 1 genotypes accounted for 38.3% (18/47), implying that domestic and wild deer could act as potential reservoirs for some human-infective genotypes. The genotypes carried by the same species in different environments are rather different. For example, six genotypes (Type IV, EbpC, EbpA, BEB6, COS-I, and COS-II) were identified in Pere David’s deer from Henan [24], and two genotypes (HLJD-V and MWC_d1) were identified in Pere David’s deer from Hubei [23], while ten genotypes including three known genotypes (HLJD-V, MWC_d1, and BEB6) and five novel genotypes (BJED-I to BJED-V) were identified in our study. Three genotypes (HLJD-V, HND-I, and BJCWD) were first identified in Chinese water deer. Contrasting our findings, zoonotic genotypes D and D-related genotypes were detected in wild Korean water deer [12]. To our knowledge, genotypes HLJD-V, BEB6, MWC_d1, and Genotype D-related genotype (BJFD) were first identified in fallow deer in this study. Compared with Pere David’s deer, Group 1 genotypes were found to be significantly higher in fallow deer (73.3%, 11/15) than in Pere David’s deer (25.0%, 6/24), implying that captive fallow deer had a higher potential risk of zoonotic *E. bieneusi* genotype transmission. Genotype D was the most frequently identified, not only in humans but also in livestock (sheep, goat, cattle, pig), companion animals (cat and dog), wild animals (wild boar, wild deer, non-human primates, tiger), and water sources worldwide [9]. The novel Genotype D-related genotype (BJFD) found in our study pointed to the evolutionary aspects of host expansion and host adaptation in Group 1 genotypes and the high-level genetic diversity of *E. bieneusi,* as the large number of samples had been examined from a different host. A genetic polymorphisms analysis at the ITS locus covering multiple hosts (human, livestock, cat, dog, deer, water) at the same locations will improve our understanding of the population genetic traits, host adaptation, and zoonotic transmission of *E. bieneusi* infections.

## 5. Conclusions

In summary, this study focused on the epidemic situation and genetic diversity of *E. bieneusi* in captive and free-ranging deer species in Beijing, China. *E. bieneusi* infection was detected in fallow deer and Chinese water deer for the first time. Based on the analysis of the TIS gene, six known genotypes and seven novel genotypes were identified with 18 (38.3%) isolates belonging to Group 1, exhibiting potential zoonotic transmission. Our results showed that the routine sequencing analysis of *E. bieneusi* strains can greatly facilitate the improved identification and management of zoonotic protozoal diseases. For public health, future studies should systematically focus on assessing the potential threats to veterinary and public health and the transmission dynamics between animal environments and human ones.

## Figures and Tables

**Table 1:** Distribution of *E. bieneusi* genotype in cervids in Beijing, China. The novel genotypes obtained in the present study are highlighted in bold.

Species	No. Tested	No. Positive (%)	Genotype (No.)	Group
Pere David’s deer	80	24 (30.0)	HLJD-V (*n* = 12), MWC_d1 (*n* = 4), BEB6 (*n* = 1), **BJED-I to BJED-V (*n* = 7)**	Group 1 (*n* = 6), Group 2 (*n* = 18)
Fallow deer	55	15 (27.3)	HLJD-V (*n* = 2), BEB6 (*n* = 2), MWC_d1 (*n* = 1), **BJFD (*n* = 10)**	Group 1 (*n* = 11), Group 2 (*n* = 4)
Sika deer	40	5 (12.5)	CGC2 (*n* = 3), JLD-XV (*n* = 2)	Group 2 (*n* = 5)
Chinese water deer	40	3 (7.5)	HLJD-V (*n* = 1), HND-I (*n* = 1), **BJCWD (*n* = 1)**	Group 1 (*n* = 1), Group 2 (*n* = 2)
Total	215	47 (21.9)	HLJD-V (*n* = 15), MWC_d1 (*n* = 5), BEB6 (*n* = 3), CGC2 (*n* = 3), JLD-XV (*n* = 2), HND-I (*n* = 1), BJED-I to BJED-V (*n* = 7), **BJFD (*n* = 10)**, **BJCWD (*n* = 1)**	Group 1 (*n* = 18), Group 2 (*n* = 29)

**Table 2:** Factors associated with the prevalence of *E. bieneusi* in cervids in Beijing, China.

Factor	Category	No. Positive/No. Tested	Prevalence % (95% CI)	*p*-Value
Species	Pere David’s deer	24/80	30.0 (19.7–40.3)	0.012
	Fallow deer	15/55	27.3 (15.1–39.4)	
	Sika deer	5/40	12.5 (1.8–23.2)	
	Chinese water deer	3/40	7.5 (−1.0–16.0)	
Gender	Male	18/92	19.6 (11.3–27.8)	0.670
	Female	27/123	22.0 (14.5–29.4)	
Living condition	Captive	18/95	18.9 (10.9–27.0)	0.525
	Free-ranging	27/120	22.5 (14.9–30.1)	

**Table 3:** Positions of nucleotide changes of known and novel genotypes of *E. bieneusi* isolates in present study.

Genotype	12	376	378	380	381	382	383	386	390	Accession No.	Reference Genotypes
genotype D	C	G	T	C	G	T	C	C	G	AF101200	Reference
Korea-WL2	C	G	T	C	G	T	C	C	G	LC436503	Reference
BJFD	-	G	T	G	T	T	C	C	A	OM876253	Novel
HLJD-V	-	G	T	C	G	T	C	C	A	OM876246	Known
BJCWD	T	-	T	G	G	T	G	-	-	OM876245	Novel
BJED-I	C	G	A	-	-	-	-	-	-	OM876247	Novel
BJED-II	C	G	T	G	G	T	C	G	T	OM876251	Novel
BJED-III	C	G	T	G	G	T	T	G	T	OM876248	Novel
BJED-IV	T	G	T	G	G	T	C	C	A	OM876249	Novel
MWC_d1	C	G	T	C	G	T	C	C	G	MF496204	Reference
MWC_d1	-	G	T	C	G	T	C	C	A	OM876242	Known
BJED-V	-	G	C	G	T	T	C	C	A	OM876243	Novel

**Table 4:** Prevalence and genotype distribution of *Enterocytozoon bieneusi* in cervids worldwide.

Country/Location	Source	Infection Rate	Genotype (No.)	Reference
China/Northern	Sika deer	13.57% (111/818)	BEB6 (84), EbpC (3), I (1), JLD-III (1), JLD-VIII (3), JLD-IX (1), JLD-XV to JLD-XXIII (17), LND-I (1)	[5]
China/Northeast	Reindeer	16.8% (21/125)	Peru6 (6), CHN-RD1 (12), CHN-RD2 to CHN-RD4 (1)	[21]
China/Hubei	Pere David’s deer	35.2% (45/128)	HLJD-V (42), MWC_d1 (3)	[23]
China/Henan	Pere David’s deer	34.0% (16/47)	Type IV (4), EbpC (4), EbpA (4), BEB6 (2), COS-I (1), COS-II (1)	[24]
China/Sichuan	Hog deer, Sambar deer, Fallow deer, Red deer, Pere David’s deer, Sika deer	24.0% (6/25)	BEB6 (4), CHS9 (1), SC03 (1)	[25]
China/Sichuan	Forest musk deer	17.04% (38/223)	SOC3 (38)	[26]
China/Northern	Red deer, Siberian roe deer	8.2% (10/122)	BEB6 (9), HLJD-VI (1)	[27]
China/Jilin	Sika deer	7.1% (23/326)	J (11), BEB6 (4), EbpC (1), CHN-DC1 (1), KIN-1 (1), JLD-1 (2), JLD-2 (2), JLD-3 (1)	[28]
China/Northeast	Sika deer, Red deer	31.9% (29/91)	BEB6 (20), HLJD-I to HLJD-IV (1), HLJD-V (5)	[29]
China/Henan and Jilin	Sika deer, Red deer	35.9% (221/615)	BEB6 (131), HLJD-I (18), EbpC (3), HLJD-IV (2), COS-I (1), EbpA (1), D (1), JLD-I (7), JLD-II (5), HND-I (4), JLD-III (2), HND-II (1), JLD-IV (6), JLD-V (2), JLD-VI (5), HND-III (1), JLD-VII (1), JLD-VIII (16), JLD-IX (1), JLD-X (1), HND-IV (1), JLD-XI (2), JLD-XII (1), JLD-XIII (1), JLD-XIV (7)	[30]
China/Beijing	Pere David’s deer, Fallow deer, Sika deer, Chinese water deer	21.9% (47/215)	HLJD-V (*n* = 15), MWC_d1 (*n* = 5), BEB6 (*n* = 3), CGC2 (*n* = 3), JLD-XV (*n* = 2), HND-I (*n* = 1), BJED-I to BJED-V (*n* = 7), BJFD (*n* = 10), BJCWD (*n* = 1)	This study
USA/Maryland	White-tailed deer	32.5% (26/80)	I (7), J (1), WL4 (11), LW1 (1), DeerEb1-DeerEb13 (13)	[22]
USA/New York	White-tailed deer	12.2% (6/49)	WL18 (2), WL19 (2), WL4 (2)	[31]
Korean/Wildlife centers	Korean water deer	53.6% (52/97)	D (29), Korea-WL1-WL6 (23)	[12]
Australia	Sambar deer *, Red deer, Fallow deer	4.1% (25/610)	D (3), J (1), Type IV (1), MWC_d1 (19), MWC_d2 (1)	[16]

*: Bold font means positive for *E. bieneusi* in corresponding study.

## Data Availability

The datasets presented in this study can be found in the article.

## References

[1] Li W., Xiao L. (2020). Ecological and public health significance of *Enterocytozoon bieneusi*. One Health.

[2] Matos O., Lobo M.L., Xiao L. (2012). Epidemiology of *Enterocytozoon bieneusi* infection in humans. J. Parasitol. Res..

[3] Didier E.S. (2005). Microsporidiosis: An emerging and opportunistic infection in humans and animals. Acta Tropica..

[4] Li W., Zhong Z., Song Y., Gong C., Deng L., Cao Y., Tian Y., Li H., Feng F., Zhang Y. (2018). Human-pathogenic *Enterocytozoon bieneusi* in captive giant pandas (*Ailuropoda melanoleuca*) in China. Sci. Rep..

[5] Tao W., Ni H., Du H., Jiang J., Li J., Qiu H., Li Y., Zhang X. (2020). Molecular detection of *Cryptosporidium* and *Enterocytozoon bieneusi* in dairy calves and sika deer in four provinces in Northern China. Parasitol. Res..

[6] Han B., Weiss L.M. (2018). Therapeutic targets for the treatment of microsporidiosis in humans. Expert Opin. Ther. Targets.

[7] Feng S., Jia T., Huang J., Fan Y., Chang H., Han S., Luo J., He H. (2020). Identification of *Enterocytozoon bieneusi* and *Cryptosporidium* spp. in farmed wild boars (*Sus scrofa*) in Beijing, China. Infect. Genet. Evol..

[8] Santin M., Fayer R. (2011). Microsporidiosis: *Enterocytozoon bieneusi* in domesticated and wild animals. Res. Vet. Sci..

[9] Li W., Feng Y., Santin M. (2019). Host specificity of *Enterocytozoon bieneusi* and public health implications. Trends Parasitol..

[10] Wang L., Xiao L., Duan L., Ye J., Guo Y., Guo M., Liu L., Feng Y. (2013). Concurrent infections of *Giardia duodenalis*, *Enterocytozoon bieneusi*, and *Clostridium difficile* in children during a cryptosporidiosis outbreak in a pediatric hospital in China. PLoS Negl. Trop. Dis..

[11] Li W., Feng Y., Xiao L. (2020). Diagnosis and molecular typing of *Enterocytozoon bieneusi*: The significant role of domestic animals in transmission of human microsporidiosis. Res. Vet. Sci..

[12] Amer S., Kim S., Han J., Na K. (2019). Prevalence and genotypes of *Enterocytozoon bieneusi* in wildlife in Korea: A public health concern. Parasites Vectors.

[13] Cong W., Qin S.Y., Meng Q.F. (2018). Molecular characterization and new genotypes of *Enterocytozoon bieneusi* in minks (*Neovison vison*) in China. Parasite.

[14] Perec-Matysiak A., Bunkowska-Gawlik K., Kvac M., Sak B., Hildebrand J., Lesnianska K. (2015). Diversity of *Enterocytozoon bieneusi* genotypes among small rodents in southwestern Poland. Vet. Parasitol..

[15] Zhang Y., Koehler A.V., Wang T., Haydon S.R., Gasser R.B. (2018). First detection and genetic characterisation of *Enterocytozoon bieneusi* in wild deer in Melbourne’s water catchments in Australia. Parasites Vectors.

[16] Yamazaki S., Motoi Y., Nagai K., Ishinazaka T., Asano M., Suzuki M. (2011). Sex determination of sika deer (Cervus nippon yesoensis) using nested PCR from feces collected in the field. J. Vet. Med. Sci..

[17] Zhang Q., Zhang Z., Ai S., Wang X., Zhang R., Duan Z. (2019). *Cryptosporidium* spp., *Enterocytozoon bieneusi*, and *Giardia duodenalis* from animal sources in the Qinghai-Tibetan Plateau Area (QTPA) in China. Comp. Immunol. Microbiol. Infect. Dis..

[18] Katoh K., Rozewicki J., Yamada K.D. (2019). MAFFT online service: Multiple sequence alignment, interactive sequence choice and visualization. Brief. Bioinform..

[19] Kumar S., Stecher G., Li M., Knyaz C., Tamura K. (2018). MEGA X: Molecular evolutionary genetics analysis across computing platforms. Mol. Biol. Evol..

[20] Cama V.A., Pearson J., Cabrera L., Pacheco L., Gilman R., Meyer S., Ortega Y., Xiao L. (2007). Transmission of *Enterocytozoon bieneusi* between a child and guinea pigs. J. Clin. Microbiol..

[21] Liu W., Nie C., Zhang L., Wang R., Liu A., Zhao W., Li H. (2015). First detection and genotyping of *Enterocytozoon bieneusi* in reindeers (Rangifer tarandus): A zoonotic potential of ITS genotypes. Parasites Vectors.

[22] Santin M., Fayer R. (2015). *Enterocytozoon bieneusi*, *Giardia*, and *Cryptosporidium* infecting white-tailed deer. J. Eukaryot. Microbiol..

[23] Xie F., Zhang Z., Zhao A., Jing B., Qi M., Wang R. (2019). Molecular characterization of *Cryptosporidium* and *Enterocytozoon bieneusi* in Père David’s deer (*Elaphurus davidianus*) from Shishou, China. Int. J. Parasitol. Parasites Wildl..

[24] Zhang Z., Huang J., Karim M.R., Zhao J., Dong H., Ai W., Li F., Zhang L., Wang R. (2015). Zoonotic *Enterocytozoon bieneusi* genotypes in Père David’s deer (*Elaphurus davidianus*) in Henan, China. Exp. Parasitol..

[25] Li W., Deng L., Yu X., Zhong Z., Wang Q., Liu X., Niu L., Xie N., Deng J., Lei S. (2016). Multilocus genotypes and broad host-range of *Enterocytozoon bieneusi* in captive wildlife at zoological gardens in China. Parasites Vectors.

[26] Song Y., Li W., Liu H., Zhong Z., Luo Y., Wei Y., Fu W., Ren Z., Zhou Z., Deng L. (2018). First report of *Giardia duodenalis* and *Enterocytozoon bieneusi* in forest musk deer (*Moschus berezovskii*) in China. Parasites Vectors.

[27] Zhao W., Wang J., Yang Z., Liu A. (2017). Dominance of the *Enterocytozoon bieneusi* genotype BEB6 in red deer (*Cervus elaphus*) and Siberian roe deer (*Capreolus pygargus*) in China and a brief literature review. Parasite.

[28] Zhang X., Cong W., Liu G., Ni X., Ma J., Zheng W., Zhao Q., Zhu X. (2016). Prevalence and genotypes of *Enterocytozoon bieneusi* in sika deer in Jilin province, Northeastern China. Acta Parasitol..

[29] Zhao W., Zhang W., Wang R., Liu W., Liu A., Yang D., Yang F., Karim M.R., Zhang L. (2014). *Enterocytozoon bieneusi* in sika deer (*Cervus nippon*) and red deer (*Cervus elaphus*): Deer specificity and zoonotic potential of ITS genotypes. Parasitol. Res..

[30] Huang J., Zhang Z., Yang Y., Wang R., Zhao J., Jian F., Ning C., Zhang L. (2017). New genotypes of *Enterocytozoon bieneusi* isolated from sika deer and red deer in China. Front. Microbiol..

[31] Guo Y., Alderisio K.A., Yang W., Cama V., Feng Y., Xiao L. (2014). Host specificity and source of *Enterocytozoon bieneusi* genotypes in a drinking source watershed. Appl. Environ. Microbiol..

[32] Yue D., Ma J., Li F., Hou J., Zheng W., Zhao Q., Zhang X., Zhu X. (2017). Occurrence of *Enterocytozoon bieneusi* in Donkeys (*Equus asinus*) in China: A Public Health Concern. Front. Microbiol..

